# Knowledge, psychological impacts, and protective behaviours during the first wave of the COVID-19 pandemic among Chinese residents in Canada with dependent school-age children: a cross-sectional online study

**DOI:** 10.1186/s12889-023-16923-x

**Published:** 2023-11-02

**Authors:** Yujia Kong, Lance Garrett Shaver, Fuyan Shi, Lixia Yang, Weiguo Zhang, Xiaoling Wei, Eleen Zhang, Sara Ozbek, Andem Effiong, Peizhong Peter Wang

**Affiliations:** 1 Division of Community Health & Humanities, Faculty of Medicine, Memorial University of Newfoundland, St. John’s, NL A1B 3V6 Canada; 2 School of Public Health, Weifang Medical University, Weifang, Shandong China; 3 Faculty of Medicine, University of British Columbia, Vancouver, Canada; 4 Department of Psychology, Ryerson University, Toronto, Canada; 5 Centre for New Immigrant Well-Being (CNIW), Markham, Canada; 6 Department of Sociology, University of Toronto Mississauga, Mississauga, Canada; 7 Dalla Lana School of Public Health, University of Toronto, Toronto, Canada; 8 School of Pharmacy, University of Waterloo, Waterloo, Canada

**Keywords:** COVID-19, Children, Immigrants, Chinese, Canada, Knowledge, Behaviours, Psychology, Implications

## Abstract

**Background:**

The purpose of this study was to describe the knowledge, protective behaviours, and psychological impact of COVID-19 on Chinese residents in Canada, as the emotional and behavioural impacts of the pandemic have not been intensively studied amongst these populations. It was important to determine whether having dependent school-age children (DSAC) aged 16 or under was associated with adverse psychological impacts amongst the Chinese residents living in the country.

**Methods:**

In April 2020, 757 eligible participants were recruited through a snowball sampling to complete an online survey related to the COVID-19 pandemic. Psychological, behavioural, and sociodemographic variables were collected and first analyzed using descriptive and univariate statistics. Multiple logistic regression analyses were performed to further confirm the observed significant associations in bivariate analyses for selected psychological outcome variables.

**Results:**

Seven hundred forty-two participants who responded to the “dependent school-age children” question were included in the analysis. Most of them identified as females (65.8%) and 77.2% included receiving a university degree or higher. There were no significant differences in COVID-19 knowledge between those living with or without DSAC. However, participants with DSAC were more likely to perceive themselves as being at greater risk of contracting COVID-19 (*p* = .023); therefore, having a higher chance of adopting protective behaviours (e.g., hand washing, sanitizing frequently or disinfecting work and living spaces (*p* < .05), elevated risks of depression (*p* = .007), and stress (*p* = .010), compared to those without DSAC.

**Conclusions:**

Predominantly, the Chinese residents in Canada with dependent school-age children were more likely to report the negative psychological impacts of the pandemic. These findings warrant further investigations that may contribute to informing key stakeholders about the identification and implementation of policies and interventions to support the needs of parents with young children, during and after the pandemic.

## Background

COVID-19 was declared a pandemic by the World Health Organization (WHO) on March 11, 2020 [1]. The outbreak evolved rapidly, with more than 80 million confirmed cases globally and a mortality rate of about 3.7% at the end of 2020 [2–4]. The first imported case of COVID-19 in Canada was recorded on January 25, 2020, with the first community transmission reported on March 1, 2020 [5]. Previous studies have found that the impact of COVID-19 on immigrant communities differed from that of local residents [6]. Before March 2020, a large proportion of COVID-19 cases in Canada were traced back to international travellers directly or indirectly linked to Wuhan, Hubei Province, China. This is where the novel coronavirus, the precursor to COVID-19, first originated [7, 8]. As Chinese immigrants were more likely to be in close contact with international travellers, the risk of infection was predominant within these populations at the earlier stages of the pandemic. Moreover, Asian immigrants in Canada are typically more socioeconomically disadvantaged compared to native-born Canadians. The disparities and inequities they experience may make them more susceptible to COVID-19 infections and related adverse health outcomes, compared to Euro-Canadians [9–11].

Chinese immigrants who were more likely to encounter travellers from COVID-19 hotspots reported experiencing a flood of negative emotions, including fear and anxiety [12]. They were often subject to discriminatory and hateful behaviours such as implicit bias, insults, attacks, and racism, generally from individuals who accused the population of instigating a worldwide pandemic [12, 13]. These issues call for more research, programs, and policies to be implemented to address the inequities and challenges experienced by Chinese immigrants living in Canada. Among research studies on knowledge, behaviours, and psychological impacts of COVID-19 on different populations, this is the first study of its kind to examine Chinese immigrants with dependent school-age children living in Canada. Furthermore, the lack of effective drugs and vaccines against COVID-19 in the early stages of the pandemic, as well as other uncertainties, led to a nationwide shutdown of schools and daycare institutions [14, 15]. While most schools provided online courses for students, the familiar structure and social environments were absent for many children and their families [16]. These disruptions were found to negatively affect behavioural and emotional health in children and their caregivers [17, 18].

This study was based on an online COVID-19 epidemiological survey with a specific focus on Chinese residents in Canada. Literature suggests that parents or guardians with dependent school-age children (DSAC, aged 16 years and under) were often highly vulnerable to negative emotions due to their increased caregiver duties during the pandemic [19], with stronger motivation to protect their families from COVID-19 than those without. This was consistent with the health belief (HB) model and protection motivational (PM) theory [20–23]. In the HB model, there are two components of a person’s characterization of health behaviour to counter the risks of a disease. The first is an understanding of the severity and susceptibility of the disease, followed by an appraisal of actions to mitigate this risk [20–23]. Although parental concerns exist amongst those who live with older children, specific health concerns as well as perceived general severity and susceptibility to disease are frequently amplified in younger children. Therefore, caregivers may be more motivated to introduce risk mitigation interventions in younger children as they have greater parental control compared to older children, who may not voluntarily comply and often possess greater social independence [20–23]. As a result, this study may provide insight into potential targeted prevention or intervention for parents and children.

The PM theory postulates that the adaptation of a protective behaviour is predicted by how individuals process such threats and act to cope with the potential harms that can arise [20–23]. The desire of parents to protect their children is frequently motivated by intrinsic or extrinsic contextual and social factors. Some of these determinants can be motivation, cultural dynamics, children’s characteristics (age, gender), the presence or absence of disability, beliefs, knowledge, and socioeconomic factors [20–23]. Accordingly, Chinese immigrant parents with younger children may be more motivated to protect them from exposure to COVID-19 than parents with older children, as these protections are critical and younger children generally do not possess the means to avoid such harms [24, 25].

Although governments have developed guidelines for school-age children, with education systems offering online courses and psychological support to minimize the impact of the pandemic on students, quality of life and mental health-related burdens persist [25–27]. The objective of this study was to describe the knowledge, behaviours, and psychological impacts of COVID-19 in a sample of Chinese immigrant parents residing in Canada with dependent school-age children (aged 16 and under).

## Methods

### Survey

An online cross-sectional survey was conducted from April 2 to April 20, 2020. Inclusion criteria were: (1) Chinese residents in North America; (2) adults over the age of 16; and (3) consent to participate in the survey.

Eligible participants were recruited through various channels, including WeChat (85.5% of participants), emails (7.7%), and through links that were posted on Chinese media websites (6.8%). Potential participants would read a brief description of the study, an assurance of anonymity, and participation rights, and give consent to participating by clicking the “agree” button. To avoid multiple submissions and to encourage truthful responses, no incentive was offered for participation, and the IP addresses of submissions were tracked. All identifying information, such as WeChat ID and IP address, was removed before analysis.

### Sampling strategy and sample size

As part of the response to the urgent call from the Canadian government, our study was carried out during the early stage of the pandemic in Canada, shortly after lockdowns were introduced and long before the COVID-19 vaccine became a reality. Canada was grappling with panic, fear, and confusion. Thus, the main goal of our project was to capture the impact of this unprecedented event full of uncertainties on people’s lives and well-being. This study was descriptive in nature and was not intended to assess pre-defined specific outcome variables. Our initial plan was to complete the survey before the pandemic ended and gain as many eligible participants as possible within approximately two weeks. While we were aware that providing incentives might be an effective means to increase the sample size, due to budget constraints and concerns about introducing unexpected biases, we decided not to offer incentives to the study participants. While we did not have the necessary information to pre-calculate the sample size, we decided to collect data from at least 500 respondents as it would meet the sample size requirements for most possible multiple regression analyses (with an alpha of 0.05 and a statistical power of 0.8).

The survey consisted of two parts. The first part collected general sociodemographic information about the subjects. The second part assessed perceptions and actions related to COVID-19, including protection, psychological impacts, knowledge of COVID-19, and an appraisal of crisis management by Canadian health authorities. Additionally, participants with DSAC were asked to describe specific actions they would take to protect their families from COVID-19. To better understand participants’ perceptions of COVID-19, the survey included questions about the virus source, transmission routes, and disease susceptibility. Moreover, the subjects were asked to rate their likelihood of being infected based on a 5-point Likert scale, from “impossible” (= 1) to “very likely” (= 5). Responses of 1 or 2 were categorized as “unlikely,” 3 as “neutral,” and 4 or 5 as “likely.” Participants also rated the psychological impacts of COVID-19 (e.g., not at ease, scared, anxious, depressed, stressed, indecisive, and confused) on a 5-point Likert scale. This is a descriptive study that compares outcomes among different groups.

### Data analysis

Descriptive analyses were conducted to report the sociodemographic characteristics of the study participants. Chi-square tests were conducted to compare the knowledge and psychological impacts of COVID-19 and the perceived likelihood of getting COVID-19 between the two groups. Missing data were not imputed. The two-sided comparison analysis used a statistically significant level of 0.05. Given the volume of information in this study, consideration was given to possible approaches to presenting the research results. A descriptive approach was used based on the empirical literature, as it allowed the topic to be examined from multiple perspectives within this one unique study. Multiple logistic regression analyses were performed to further confirm the observed associations in bivariate analyses.

Data analyses were performed using SPSS statistical software (version 21.0, IBM Company, Armonk, NY, USA, 2014). The bar chart was drawn with STATA software (version 16.0, Stata Corp., USA, 2019).

This study was approved by the Health Research Ethics Board, Memorial University of Newfoundland, file number 20,201,772-ME. All methods were performed under the relevant guidelines and regulations.

## Results

A total of 757 eligible participants completed the survey. However, 742 people (258 males and 484 females) responded to the question of whether they have a child 16 years of age or under, thus including them in the final data analysis. About 39.4% (292/742) of the respondents stated that they had at least one child 16 years of age or under, Table 1.

**Table 1:** Characteristics of study participants

Characteristics^a^		Participants with children ≤ 16 years, n (%)	
		Yes	No
Provinces	Ontario	245 (39.4)	377 (60.6)
	British Columbia	22 (38.6)	35 (61.4)
	Other provinces	25 (39.7)	38 (60.3)
Gender	Male	97 (37.6)	161 (62.4)
	Female	195 (40.3)	289 (59.7)
Age groups	≤ 34	17 (12.0)	125 (88.0)
	35–54	228 (58.8)	160 (41.2)
	55+	46 (21.8)	165 (78.2)
Birthplace	Mainland China	286 (39.8)	433 (60.2)
	Other places	6 (26.1)	17 (73.9)
Living in Canada	No more than 5 years	69 (42.9)	92 (57.1)
	More than 5 years	223 (38.4)	357 (61.6)
Marital status	Married/ Common law	259 (46.5)	298 (53.5)
	Other	33 (17.8)	152 (82.2)
Education	High school or less	20 (51.3)	19 (48.7)
	College/ University	177 (40.2)	263 (59.8)
	Master’s degree or higher	93 (36.0)	165 (64.0)
Health worker	No	266 (38.7)	422 (61.3)
	Yes	26 (49.1)	27 (50.9)
Living status	Living alone	3 (4.0)	72 (96.0)
	Not living alone	289 (43.3)	378 (56.7)
Employment status	Employment	97 (34.3)	186 (65.7)
	Retire	28 (31.5)	61 (68.5)
	Other	167 (45.1)	203 (54.9)
Income satisfaction	Dissatisfied	51 (37.0)	87 (63.0)
	Neutral	126 (43.4)	164 (56.6)
	Satisfied	105 (36.3)	184 (63.7)
Health status	Poor	9 (29.0)	22 (71.0)
	Average	77 (39.9)	116 (60.1)
	Good	201 (39.6)	306 (60.4)
Total	742	292 (39.4)	450 (60.6)

^a^ System-missing was classified into the category “Other” if such a response option category existed for that survey item

Participants’ knowledge of the COVID-19 pandemic, specifically regarding the source, transmission channel, and disease susceptibility, was displayed in Table 2. While the majority of participants actively sought COVID-19-related information through various channels, there were a significant number of participants who had some misconceptions about COVID-19. Specifically, 36.1% of participants believed that the virus might originate from a high-level biosafety laboratory, though only 6.2% of them considered COVID-19 as a type of biochemical weapon. Almost all participants agreed that physical contact and respiratory droplets (e.g., saliva) are important routes for virus transmission. Furthermore, 72.9% of participants were aware of airborne transmission. Although more than 60% of participants agreed that the elderly and immunosuppressed were the vulnerable populations, more than half of the participants also agreed that “in general, all populations are susceptible to COVID-19” (note that these were not mutually exclusive options). As shown in Table 2, participants with and without DSAC did not differ in their COVID-19 knowledge. However, they were different in perceived likelihood of contracting COVID-19, *χ*^2^ = 7.513, *p* = .023. Specifically, those with DSAC were more likely to respond “likely” rather than “unlikely” to their chances of getting COVID-19, relative to those without children 16 and under (Table 3).

**Table 2:** Knowledge of COVID-19

Knowledge of COVID-19	Total n (%)	Participants with children ≤ 16 years, n (%)		χ2	*p*
		Yes	No		
**K1. Virus source**^**a**^				3.382	0.338
1. Wild animals	280 (37.7)	102 (34.9)	178 (39.6)		
2. High bio-safety laboratory	268 (36.1)	117 (40.1)	151 (33.6)		
3. A kind of biological weapon	46 (6.2)	18 (6.2)	28 (6.2)		
4. Other	148 (19.9)	55 (18.8)	93 (20.7)		
**K2. Transmission route**					
1. Airborne	541 (72.9)	223 (76.4)	318 (70.7)	2.916	0.091
2. Contact transmission	698 (94.1)	276 (94.5)	422 (93.8)	0.175	0.752
3. Droplet transmission (e.g., saliva)	726 (97.8)	285 (97.6)	441 (98.0)	0.132	0.798
4. Oral-fecal transmission	429 (57.8)	158 (54.1)	271 (60.2)	2.713	0.110
5. Other	69 (9.3)	22 (7.5)	47 (10.4)	1.778	0.198
**K3. Susceptible population**					
1. Older people (older than 50)	445 (60.0)	179 (61.3)	266 (59.1)	0.354	0.592
2. Teenagers	84 (11.3)	28 (9.6)	56 (12.4)	1.438	0.239
3. People who are immune suppressed	506 (68.2)	205 (70.2)	301 (66.9)	0.898	0.375
4. All people are equally susceptible	401 (54.0)	153 (52.4)	248 (55.1)	0.525	0.498
Total	742 (100.0)	292 (39.4)	450 (60.6)		

K1: “In your opinion, what is the most likely source of SARS-COV-2 virus? Choose one only”

K2: “In your opinion, can COVID-19 be transmitted through the following routes? Choose all apply”

K3: “In your opinion, which population is susceptible to COVID-19 infection? Choose all apply”

^a^Responses to the question on virus source were mutually exclusive, so only one Chi-square test was performed, whereas the responses to questions on transmission route and susceptible populations were not necessarily mutually exclusive so Chi-square tests were performed for each row

**Table 3:** Perceived likelihood of getting COVID-19

Perceived likelihood of getting COVID-19	Participants with children ≤ 16 years, n (%)		*χ*2	*p*
	Yes	No		
			7.513	0.023^*^
Unlikely	96 (35.7)	196 (46.0)		
Neutral	126 (46.8)	173 (40.6)		
Likely	47 (17.5)	57 (13.4)		
Total	292 (39.4)	450 (60.6)		

^*^ Indicates statistically significant difference at the level of 0.05

In terms of the psychological impact of the pandemic, 50% of the participants felt not at ease, nearly half felt anxious and stressed, and more than one-third felt scared and confused (Table 4). Our bivariate analysis results suggest more individuals with the DSAC reported negative emotions such as not being at ease (*χ*2 = 6.077, *p* = .047*)*, depressed (*χ*2 = 10.033, *p* = .007), and stressed (*χ*2 = 9.253, *p* = .010).

**Table 4:** Psychological feelings towards COVID-19 between participants with and without children ≤ 16 years

Feeling	Total n (%)	Participants with children ≤ 16 years, n (%)		*χ*2(2)	*p*
		Yes	No		
**At ease**				6.077	0.047*
Disagree	358 (54.7)	143 (57.4)	215 (53.0)		
Neutral	211 (32.2)	67 (26.9)	144 (35.5)		
Agree	86 (13.1)	39 (15.7)	47 (15.7)		
**Scared**				2.155	0.344
Disagree	180 (25.1)	69 (24.2)	111 (25.6)		
Neutral	254 (35.4)	94 (33.0)	160 (37.0)		
Agree	284 (39.6)	122 (42.8)	162 (37.4)		
**Anxious**				3.269	0.194
Disagree	170 (23.8)	63 (22.3)	107 (24.8)		
Neutral	223 (31.2)	81 (28.6)	142 (32.9)		
Agree	321 (45.0)	139 (49.1)	182 (42.2)		
**Depressed**				10.033	0.007*
Disagree	273 (43.2)	97 (37.9)	176 (46.8)		
Neutral	191 (30.2)	74 (28.9)	117 (31.1)		
Agree	168 (26.6)	85 (33.2)	83 (22.1)		
**Stressed**				9.253	0.010*
Disagree	178 (25.9)	65 (23.4)	113 (27.6)		
Neutral	194 (28.2)	66 (23.7)	128 (31.2)		
Agree	316 (45.9)	147 (52.9)	169 (41.2)		
**Indecisive**				3.827	0.149
Disagree	255 (38.9)	96 (36.0)	159 (41.0)		
Neutral	225 (34.4)	89 (33.3)	136 (35.1)		
Agree	175 (26.7)	82 (30.7)	93 (24.0)		
**Confused**				3.940	0.139
Disagree	205 (31.4)	72 (27.6)	133 (33.9)		
Neutral	202 (30.9)	80 (30.7)	122 (31.1)		
Agree	246 (37.7)	109 (41.8)	137 (34.9)		
Total	742 (100)	292 (39.3)	450 (60.6)		

* Indicates statistically significant difference at the level of 0.05

**Fig. 1:**
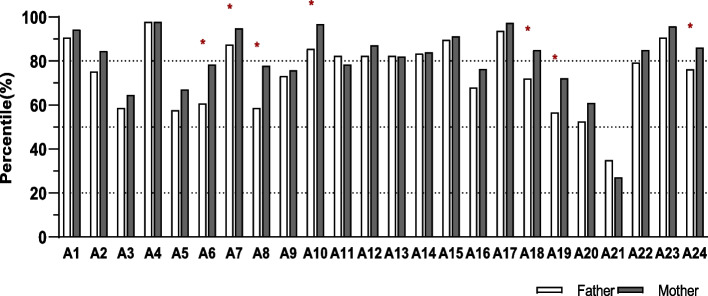
Reported protective behaviours during the COVID-19 pandemic among different gender subgroups (fathers and mothers) of parents of children 16 years and under (*n* = 292). A1: Cover sneeze with elbow or tissue paper, A2: Avoid touching nose, mouth, and eyes, A3: Use serving utensils for shared food during mealtime, A4: Wash hands frequently (using soap), A5: Sanitize hands frequently (using hand sanitizer), A6*: Disinfect home/work surroundings, A7*: Wear mask in public, A8*: Wear gloves in public, A9: Keep the room well ventilated, A10*: Keep social distance (at least 2 m), A11: Change greeting procedures (e.g., avoid handshakes and hugs), A12: Self-isolation when experiencing cold/flu/COVID-19 symptoms, A13: Report to relevant organization when COVID symptoms appear, A14: Self isolation for 14 days after encounter with presumptive or confirmed COVID-19 case, A15: Reduce using public transportation, A16: Work or study at home, A17: Cancel group activities, A18*: Stock non-perishable food items and supplies, A19*: Purchase dietary supplements and/or medicine, A20: Improve diet quality, A21: Ask children to stay home (prior to school closure), A22: Ask children to avoid group activities, A23: Educate children about preventative behaviors, A24*: Ask children to study from home. Bar with * and items in bold indicates statistically significant difference between parents at the level of 0.05. * Indicates statistically significant difference at the level of 0.05

In terms of protective behaviours against COVID-19, individuals with DSAC have largely adopted practices such as covering a sneeze with elbows or tissue paper, sanitizing hands frequently, limiting the use of public transportation, avoiding, or cancelling group activities, and educating children about preventative behaviours (Fig. 1).

The analysis (Fig. 1, A7) showed that mothers outperformed fathers in terms of wearing masks in public areas (*p* = .034) and maintaining social distancing (*p* = .001). Almost all participants claimed to wash their hands frequently, while only two-thirds used alcohol-based hand sanitizer. More than 75% of mothers and roughly 60% of fathers said they would disinfect their surroundings, (*χ*^2^(1) = 10.13, *p* = .002). A similar trend was observed for wearing gloves in public, where mothers fared better, *χ*^2^(1) = 11.72, *p* = .001. During the early stages of COVID-19, 80.6% of individuals with DSAC said they would stock up on non-perishable food and supplies, and 67.1% said they would buy dietary supplements or medicines. Compared to fathers, significantly more mothers said they would stock up on food and supplies (85.1% vs. 72.2%, *χ*^2^(1) = 7.02, *p* = .011) and buy dietary supplements or medicines (72.3% vs. 56.7%, *χ*^2^(1) = 7.15, *p* = .008). Before the school closures, very few parents stated that they would ask their children to stay at home (35.1% for fathers and 27.2% for mothers); however, 83.2% of them stated that they asked their children to avoid group activities.

Seven of the protective factors showed statistically significant differences in mothers adopting far more public health measures than fathers. This data shows the differential behaviours of parents and the psychological impact when dealing with certain circumstances (COVID-19 pandemic).

The statistically significant associations as shown in Table 4 remain for variables depressed and stressed in multiple logistic regression analysis (not shown). Parents with DSAC have higher odds of having depression compared with those without DSAC while controlling for other respondent’s characteristics (OR = 1.44 (95% CI 1.04–2.01)) (Table 5).

**Table 5:** Psychological impact of having underage children, results from multiple logistic regression analyses (*n* = 742)

Variables	No at Ease	Depressed	Stressed
	aOR (95%CI)	aOR (95%CI)	aOR (95%CI)
Province			
Ontario (Ref)	-	-	-
British Columbia	1.13(0.60–2.11)	0.77(0.41–1.42)	0.87(0.45–1.69)
Other provinces	0.89(0.49–1.62)	1.43(0.76–2.69)	0.92(0.48–1.77)
Gender			
Male (Ref)	-	-	-
Female	0.91(0.64–1.29)	0.83(0.58–1.19)	1.00(0.68–1.48)
Age	1.21(0.88–1.66)	1.42(1.03–1.99)^*^	1.27(0.90,1.81)
Birthplace			
Mainland China (Ref)	-	-	-
Other places	0.66(0.24–1.81)	1.44(0.50–4.13)	1.0810.33–3.56)
Living in Canada ≥ 5 years	0.81(0.52–1.27)	0.91(0.58–1.44)	1.41(0.87–2.28)
Marital status			
Married/Common law (Ref)	-	-	-
Other	0.86(0.53–1.40)	1.25(0.76–2.08)	1.41(0.81–2.47)
Education	0.84(0.62–1.16)	1.20(0.87–1.64)	1.06(0.76–1.50)
Health worker	1.06(0.56–2.02)	1.93(0.95–3.92)	0.83(0.41–1.69)
Not living alone	1.91(1.01–3.58)^*^	0.95(0.51–1.78)	1.04(0.51–2.12)
Employment status			
Employment (Ref)	-	-	-
Retire	0.58(0.31–1.10)	0.80(0.41–1.57)	0.48(0.24–0.95)^*^
Other	0.92(0.63–1.34)	0.94(0.64–1.38)	0.98(0.64–1.49)
Income satisfaction	0.59(0.46–1.75)^*^	0.74(0.58–0.93)^*^	0.61(0.47–0.80)^*^
Health status	0.69(0.50,0.96)^*^	0.73(0.53 − 0.10)^*^	0.74(0.52–1.07)
Having child under 16	1.04(0.73–1.48)	1.53(1.06–2.21)^*^	1.36(0.92–2.03)

^*^ *P* < .05

## Discussion

In response to the COVID-19 epidemic, the Canadian government has implemented various policies and measures to promote epidemic-related research, such as CIHR 2020, supported by the New Frontiers in Research Fund (NFRF). Due to the high contagiousness and fatality rate of COVID-19, all schools and childcare institutions in Canada were closed during the onset of the outbreak [28–30]. Although the government has developed guidelines for school-age children, with education systems offering online courses and psychological support to minimize the impact of the pandemic on students and their families, some of these measures have had unintended consequences [31].

This research was part of a larger project focusing on the Chinese communities in Canada. It was conducted during the second month of the nationwide lockdown due to the COVID-19 pandemic. During this period, a general state of confusion and panic had expanded caused by the coronavirus both within and outside Canada [31–33]. This is the first comprehensive study on the knowledge, behaviour, and psychological impacts related to COVID-19 amongst Chinese residents in Canada. Furthermore, it is also one of the only preliminary studies focusing on the Chinese caregivers (primary parents) of dependent school-age children. These findings may help governments identify and support the unique needs of parents with young children.

Our study has shown that the efforts taken by the Canadian government to promote knowledge related to COVID-19 through various channels and methods have been successful in the target population of this study, as most participants had basic knowledge relating to the virus, such as transmission routes and main prevention measures [34]. Nonetheless, parents with and without DSAC were found to hold few misconceptions about the pandemic. These findings resemble those reported in previous literature [35–37]. During the initial stages of the pandemic, many participants panicked and actively sought out as much information as possible from reliable and unreliable sources [38, 39]. More than half of our participants believed that all people were equally susceptible to COVID-19, which was possibly a reflection of people’s fear of the pandemic [40, 41]. When participants were asked about their perceived likelihood of getting infected with COVID-19, those who had DSAC stated that they were more likely to be infected, suggesting a greater degree of pessimism. As mentioned in previous literature, parents of minors are usually more sensitive to major external events, which are more likely to elicit negative emotions such as stress and anxiety [42, 43].

Our results suggest that individuals with DSAC did show various levels of negative psychological emotions [44–46], specifically stronger negative psychological impacts. Similar findings have since been observed in other populations of parents outside of Canada [44–46]. Considering that COVID-19 has been the most serious global infectious disease outbreak in the past century [47, 48], persistent fears about the pandemic and uncertainty about the future inevitably lead to negative emotions.

The trends revealed in this study are consistent with previous literature on other outbreaks of infectious diseases [48–52]. Children under the age of 16 often require more companionship and support from families and friends than older children. Caregivers, particularly parents, must devote significant time and energy to their adolescents [53]. The innate behaviours of children and the necessity for social interaction during development present unique challenges to social distancing and isolation. These behaviours and needs may contribute to their parents’ feelings of unease, depression, and stress. Keeping children safe from COVID-19 can present a plethora of challenges and be mentally taxing on caregivers. Other negative aspects of the pandemic, such as income loss and a lack of normal family activities, may also have a greater impact on parents and guardians with young children. However, further exploration of the mechanism behind the observed association is warranted.

Despite their pessimistic emotions, most parents with dependent school-age children were still willing to actively respond to the WHO’s protective-behaviour guidance on effective prevention of COVID-19 during the pandemic. They were also willing to implement corresponding health-protective behaviours.

Compared with other ethnic groups in western countries, Chinese immigrants were more likely to use masks in public places to help prevent infection during the beginning of the pandemic [54]. This behaviour may have been inspired by the quick, large-scale control of COVID-19 spread using effective measures, including wearing masks, as demonstrated in China [55–57]. Most parents undertook protective behaviours to protect their family members during the pandemic, including mask-wearing, social distancing, limiting group activities, limiting the use of public transit, educating their children about preventive behaviours, and stocking up on food and supplies. We also revealed gender differences, with mothers more likely to comply with certain protective behaviours compared to fathers.

The study’s strengths lie in its novelty as it appears to be one of the first research endeavours that examines the knowledge, protective behaviours, and psychological impact of COVID-19 specifically among Chinese residents in Canada with dependent school-age children. By focusing on this specific population, the study fills a research gap and provides valuable insights into their unique experiences and challenges during the pandemic.

Another strength is the timeliness of data collection, as the study was conducted during the early stage of the COVID-19 pandemic in April 2020. This allowed for capturing the participants’ experiences and emotions during a critical period when the pandemic was rapidly evolving, and uncertainty was high. The data collected reflects the true feelings of the participants at that specific time, offering a snapshot of their psychological impact.

Furthermore, the use of an anonymous survey is an additional strength. Anonymity in the online survey reduces the potential influence of social desirability bias, encouraging participants to provide honest and candid responses. By protecting their identities, the study mitigates potential biases and enhances the reliability and validity of the collected data, providing a more accurate representation of participants’ thoughts, feelings, and behaviours related to COVID-19.

While this study was conducted during the COVID-19 pandemic, which has now transitioned from its emergency phase, and cannot be replicated, the findings hold important implications for future similar situations. The insights gained from this study can inform preparedness strategies and interventions for future outbreaks or pandemics. Additionally, while our study focused on Chinese immigrants, the results are expected to be relevant to other populations as well. The psychological impacts, protective behaviours, and knowledge gaps highlighted in this study can help guide public health efforts and tailor interventions across diverse communities facing similar challenges.

There were several limitations to our research. First, the online snowball sampling procedure, a feasible recruitment method during the pandemic, might be restricted in recruiting a representative sample. The sample might not well represent the Chinese immigrant population in Canada. Furthermore, people who participated in the survey were likely more concerned about the pandemic, therefore, a bias in selection may exist. In addition, the cross-sectional nature of the survey does not inform the longitudinal changes in participants’ beliefs, behaviours, and psychological impacts over time. Moreover, the study distinguished between participants who claimed they had children 16 and under and those who said they did not. This can be interpreted as having children over 16 or not having children at all, which could have led to heterogeneity in response. As this study was derived from a general survey, we did not ask about the number of children and their ages for each participant. Parents’ or guardians’ challenges were likely to vary depending on their children’s age. Finally, as the current study is descriptive, the observed associations are subject to confounding and need to be further confirmed in future studies.

## Conclusions

This study was able to determine that Chinese caregivers with dependent school-age children are more prone to negative emotions during the COVID-19 pandemic than those without. The social connection needs of young children might make social isolation and distancing exceptionally challenging for this age group compared to other age groups. The difficulties in teaching children to isolate and maintain social distance may have exacerbated the anxiety, frustration, and stress of parents who are obligated to keep them safe. Despite these negative psychological impacts, most parents reported taking numerous measures to protect themselves and their families. Gender differences were observed for some measures and activities.

## Data Availability

The datasets analyzed during the current study are available from the corresponding author on reasonable request.

## Abbreviations

CIHR: The Canadian Institutes of Health Research
COVID-19: Coronavirus disease
HB: Help Belief model
IBM: International Business Machines
LLC: Limited NFRP:New Frontiers in Research Fund
NY: New York
PM: Protection Motivational theory
SPSS: Statistical Product and Service Solutions
STATA: Statistics and Data
USA: United States of America
